# Effect of breastfeeding and maternal characteristics on diarrhoea morbidity among children aged 0-2 years in Namibia

**DOI:** 10.4314/ahs.v24i2.33

**Published:** 2024-06

**Authors:** Opeoluwa Oyedele

**Affiliations:** 1 Department of Computing, Mathematical and Statistical Sciences, School of Science, University of Namibia, Windhoek, Namibia; 2 Department of Environmental Health, Faculty of Health Sciences, Nelson Mandela University, Gqeberha, South Africa

**Keywords:** Breastfeeding, diarrhoea, log-binomial model, maternal characteristics, child diarrhoea morbidity, Namibia

## Abstract

**Background:**

Despite it being treatable and preventable, morbidity from diarrhoeal disease still remains one of the leading killers of young children in developing countries.

**Objectives:**

To examine the effect of breastfeeding and maternal characteristics on diarrhoea morbidity among 0-2 years old children in Namibia.

**Methods:**

A quantitative cross-sectional study design with a multivariable log-binomial model was used to examine the effect of breastfeeding and maternal characteristics on diarrhoea morbidity among 0-2 year old children from data collected from the 2013 NDHS.

**Results:**

Breastfeeding and maternal related characteristics such as breastfeeding status, sharing toilet facilities with other households, total children ever born, health insurance cover and main language spoken in home had lower risks on child diarrhoea morbidity, while characteristics such as type of place of residence, highest educational level, electricity & refrigerator in the household, religion, wealth index, type of mosquito bed net(s) slept under last night, mother's age at first birth, current age of child, child's residency and drugs taken for intestinal parasites in last 6 months had higher risks and region had mixed risks.

**Conclusions:**

Since studies have shown that the possibility of reducing the risk of morbidity related to diarrhoeal infections in children requires well-informed parents, all relevant organizations and governmental ministries that deals with health services and children's well-being should make use of mass media like radio and television to constantly spread consistent messages on breastfeeding and advocate for better implementation of sanitation and hygiene practices among mothers with children aged 0-2 years, especially in rural and poorest areas of the Kavangos (East/West) and Caprivi/Zambezi regions.

## Introduction

Children's health is very important and crucial during infancy. While protecting and improving the health of children is a fundamental importance, a great deal of work still remains to further improve the health outcomes for children. According to World Health Organization [WHO]^1^, more than half of children deaths are due to conditions that could easily be prevented or treated given the access to health care and improvements to their quality of life. Among the biggest causes of under-five children deaths is diarrhoea morbidity. Diarrhoea is when stools or bowel movements are loose and watery^2^. It can be described as the body's way of ridding itself of germs, with most episodes lasting a few days to a week, and can occur with fever, nausea, vomiting, cramps, dehydration, and even rashes^3^. It causes death by depleting body fluids resulting in profound dehydration, which can have a detrimental impact on childhood growth and cognitive development^4^. Despite the availability of a simple treatment solution, diarrhoea morbidity still remains one of the leading killers among under-five children globally. In 2017, diarrhoea morbidity accounted for approximately 8% of all deaths among children under the age of 5 years worldwide, which loosely translated to over 1400 young children dying each day or about 525000 children a year^5^. Globally, this death rate due to diarrhoea morbidity was further estimated to be more than 1 in 10 child deaths, i.e., about 800000 deaths each year^5^.

Infant mortality from diarrhoea morbidity has sharply declined in the last few decades throughout Europe and also in some rich Asian countries and high-income countries. In these countries, the death rates have been very low, below 1 per 100000 cases per year, while in low-income countries the rates have been estimated to be higher than 300 per 100000 cases per year, especially, among the poorest countries like Madagascar, Chad and the Central African Republic, with few(er) resources and less robust health infrastructure system^6^. In his study, Dadonaite^6^ further iterated that diarrhoeal diseases are the third leading cause of child mortality globally, falling just behind pneumonia and pre-term birth complications, with the death rate from diarrheal diseases highest among the world's poorest countries including Africa and Southeast Asia countries (with about 90% of diarrhoea deaths). Getahun & Adane^7^ pointed out that diarrhoea morbidity was common in the developing world due to the prevalence of unsafe drinking water, inadequate sanitation facilities, poor hygiene practices, malnutrition, undernutrition, stunting, lack of access to essential health treatment, lack of household water treatment and unsafe water storage, with UNICEF^8^ concluding that children under the age of five years in these countries were, on average, 20 times more likely to die from diarrheal diseases associated with poor water, sanitation and hygiene than from violence in conflict. This further makes diarrhoea morbidity among children under-five years one of the significant public health concerns in developing countries and an important cause of morbidity and mortality amongst children in low- and middle-income countries like Namibia. In Namibia, the national prevalence of diarrhoea morbidity stood at 17%, i.e., 40.2 per 1000 live births, and it was responsible for 5% of all deaths in children under-five years, making it the second leading cause of child death in the country^5^,^9^.

**Figure 1:**
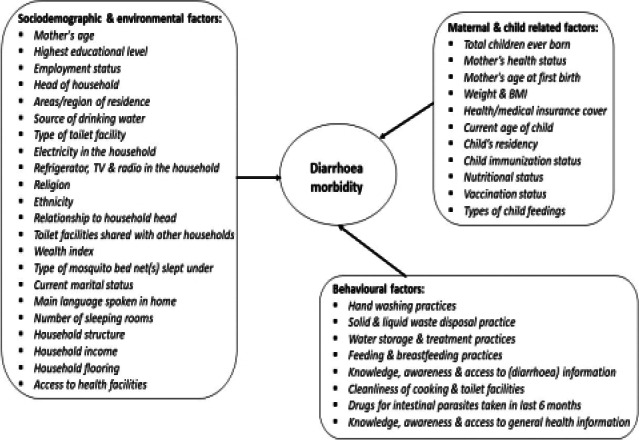
Conceptual model for some of the causes of child diarrhoea morbidity

Although various studies have indicated that epidemiologic factors that contribute to the occurrence of diarrhoea morbidity were complex, factors such as residential area, household structure, unemployment, household income, mother or caregiver age, number of people per household, access to information, type of toilet facilities, access to safe drinking water, child immunisation status, nutritional status and number of sleeping rooms have been conveyed to contribute to diarrhoeal prevalence^9^ as illustrated in Figure 1. Moreover, findings by UNICEF^10^ revealed that infants were at greater risk of death due to diarrhoea morbidity and other infections when they are only partially breastfed or not breastfed at all. This was due to the fact that breastfeeding is very vital to a child's lifelong health and reduces costs for health facilities and families, with breastfeeding within the first hour of birth protecting new born babies from infections and saving their lives. According to WHO^14^, breastfeeding is one of the most effective ways to ensure child health and survival. To be precise, breastmilk is the ideal food for infants as it is safe, clean and contains antibodies which help protect against many common childhood illnesses^14^. It provides all the energy and nutrients that the infant needs for the first months of their lives and continues to provide up to half or more of a child's nutritional needs during the second half of their first year, and up to one-third during the second year of their life^14^. UNICEF^10^ further pointed out that ‘exclusive breastfeeding saves children's lives as its benefits help keep babies healthy in their first days and last well into their adulthood’, while Dadonaite^6^ disclosed that breastfeeding allows for the transfer of maternal immunity to the child.

Furthermore, exclusive breastfeeding until the age of 6 months has also been reported as one of the cost-effective measures to help prevent acute diarrhoea morbidity in children under-five years old in developing countries^7^, to which the WHO and UNICEF recommended the early initiation of breastfeeding within one hour of birth of the child. However, over the past two decades, nearly two out of three infants are not (exclusively) breastfed for the recommended six months of their lives^14^. In developing countries, under-five children that were not breastfed were six times more likely to die from diarrhoea morbidity and infectious diseases in the first two months of their lives^6^, while exclusively breastfed babies have often been reported to have less diarrhoea morbidity^2^. Hence, morbidity from diarrhoeal disease still remains high in these countries due to insufficient and lack of (exclusive) breastfeeding, poor sanitation and hygiene among others factors^9^ and Namibia is not an exception to this. Thus, the main objective of this study was to examine the effect of breastfeeding and maternal characteristics on diarrhoea morbidity among 0-2 year old children in Namibia. Findings from this study can provide useful insights for evidence-based health policies and programmes on diarrhoea morbidity among children in the country, thereby further aiding in the effective allocation and utilization of public health programs and resources in the prevention of diarrhoea mortality among children within the country.

## Methods

### Study design and data collection

This study followed a quantitative cross-sectional study design using data obtained from the 2013 Namibia Demographic and Health Survey (NDHS), the latest of its kind. The Demographic and Health Survey (DHS), funded by the United States Agency for International Development (USAID), was part of the ‘worldwide measure DHS programs designed to collect information on fertility, family planning, infant and child mortality, maternal and child health, nutrition, domestic violence, and knowledge and prevalence of human immunodeficiency virus/acquired immunodeficiency syndrome and other non-communicable diseases’^11^. The NDHS has been conducted four times (1992, 2000, 2006/2007 and 2013) in collaboration with the Ministry of Health and Social Services (MoHSS) in Namibia. The 2013 NDHS serves as a periodic update of the demographic and health situation in Namibia, with the study initiated in April 2012 and the data collection aspect carried out from May to September 2013^11^.

### Study population and sampling strategy

The sampling design used in the 2013 NDHS was a stratified sample selected in two stages for all the then 13 administrative regions in Namibia. In the first stage, 554 enumeration areas (269 in urban areas and 285 in rural areas) were selected with a stratified probability proportional to size selection from the sampling frame^11^. The size of an enumeration area was ‘defined according to the number of households residing in the enumeration area, as recorded in the 2011 population and housing census of Namibia. Stratification was achieved by separating each region into urban and rural areas, resulting into 26 sampling strata (13 rural strata and 13 urban strata). Samples were later selected independently in every stratum, with a predetermined number of enumeration areas selected’^11^. In the second stage, a fixed number of 20 households were selected in every urban and rural cluster according to equal probability systematic sampling^11^.

More detailed information about the sampling methods and the entire survey can be found in the 2013/14 NDHS report, freely available online on the DHS website. Since children aged over two years would not be expected to be currently breastfed, the inclusion criteria for this study were all children aged 0-2 years (in the breastfeeding age range) whose mothers participated and provided information for them in the 2013 NDHS. Children with incomplete, non-response or missing information were excluded from this study.

### Statistical analysis

Pearson's chi-square test was performed to examine the association between the breastfeeding and maternal characteristics and diarrhea morbidity among children aged 0-2 years. The breastfeeding related characteristics considered in this study was the child's breastfeeding status, while the maternal related characteristics were the mother's age, region, type of place of residence, highest educational level, source of drinking water, type of toilet facility, electricity and refrigerator in the household, religion, sharing toilet facilities with other households, wealth index, type of mosquito bed net(s) slept under last night, main language spoken in home, mother's age at first birth, total children ever born, health insurance cover, current age of child, child's residency and drugs taken for intestinal parasites in last 6 months. The individual children considered in this study were identified from the NDHS as per the inclusion criteria for this study (all children aged 0-2 years, in the breastfeeding age range, whose mothers participated and provided information for them in the 2013 NDHS). The responses to questions such as ‘Did you ever breastfeed?’ and ‘Are you still breastfeeding (NAME)?’ as captured in the NDHS data were used to determine the child's breastfeeding status in this study. Similarly, the responses to the ‘Has (NAME) had diarrhoea in the last 2 weeks?’ question as captured in the NDHS data were used to determine the child's diarrhoea morbidity status in this study. More detailed information about these characteristics as well as the remaining characteristics considered in this study can be found in the 2013/14 NDHS report, freely available online on the DHS website.

Furthermore, the effect of the breastfeeding and maternal characteristics on child diarrhoea morbidity was determined using a multivariable log-binomial model. A log-binomial model is similar to the logit model^12^, except the log-binomial model uses a log-link function to connect a set of predictor variables (X) on a single dichotomous outcome (Y), while the logit model uses a logit link function. Also, a log-binomial model naturally offers the description of the relationship between and in terms of relative risks, rather than odds ratios. Relative risk provides the likelihood of an event based on some exposure^15^. If π is the probability of an event in the exposed group, then the relative risk (RR) can be estimated as


(1) RR=π/(1−π)

where *(1- π)* is the probability of an event not in the exposed group. In this study, (X) was the breastfeeding and maternal characteristics while (Y) was the children's diarrhoea morbidity status. Significant characteristics (with p-value<0.05 and p-value<0.10) from the chi-square tests were used in the fitted multivariable log-binomial model. The statistical analyses were performed using the R programming language (version 4.1.2).

### Ethical considerations

Ethical clearance for this study was obtained from the University of Namibia decentralized ethics committee. As this study was a secondary data analysis of publicly accessible survey data from the DHS program, this study did not require participant consent. The author completed the user's agreement and was granted the 2013 NDHS dataset from the DHS website. No separate permission was required for data usage and publication. Additionally, this study followed all ethical standards for research without dirct contact with human or animal subjects as there were no names of persons or household addresses recorded in the DHS data.

## Results

As per the inclusion criteria of this study, described under the methodology section of this study, a total of 3014 children aged 0-2 years were considered, out of which 674 (22.36%) had diarrhoea morbidity and 2340 (77.64%) did not. Tables 1 to 2 shows the percent distribution of the breastfeeding and maternal characteristics across child diarrhoea morbidity. From Table 1, 1331 (44.16%) children were currently breastfeeding, while 1683 (55.84%) were not. Out of the 674 children who had diarrhoea morbidity, 319 were currently breastfeeding, while 355 were not currently breastfeeding.

**Table 1:** Percent distribution of breastfeeding and child diarrhoea morbidity

	Had no diarrhoea morbidity		Had diarrhoea morbidity		Total		P-value
	Count	%	Count	%	Count	%	
**Breastfeeding status**							0.066**
Currently breastfeeding	1012	33.58	319	10.58	1331	44.16	
Not currently breastfeeding	1328	44.06	355	11.78	1683	55.84	
Total	2340	77.64	674	22.36	3014	100	

** Significant at a 10% level of significance

**Table 2:** Percent distribution of maternal characteristics and child diarrhoea morbidity

	Had no diarrhoea morbidity		Had diarrhoea morbidity		Total		P-value
	Count	%	Count	%	Count	%	
**Mother's age (in years)**							0.047*
<20	199	6.60	80	2.65	279	9.26	
20-34	1663	55.18	472	15.66	2135	70.84	
35-44	448	14.86	115	3.82	563	18.68	
45-54	30	1.00	7	0.23	37	1.23	
Total	2340	77.64	674	22.36	3014	100	
**Region**							<0.001*
Caprivi	139	4.61	100	3.32	239	7.93	
Erongo	186	6.17	27	0.90	213	7.07	
Hardap	178	5.91	20	0.66	198	6.57	
//Karas	184	6.10	33	1.09	217	7.20	
Kavango	181	6.01	115	3.82	296	9.82	
Khomas	185	6.14	54	1.79	239	7.93	
Kunene	204	6.77	44	1.46	248	8.23	
Ohangwena	232	7.70	64	2.12	296	9.82	
Omaheke	172	5.71	42	1.39	214	7.10	
Omusati	164	5.44	60	1.99	224	7.43	
Oshana	135	4.48	25	0.83	160	5.31	
Oshikoto	174	5.77	42	1.39	216	7.17	
Otjozondjupa	206	6.83	48	1.59	254	8.43	
Total	2340	77.64	674	22.36	3014	100	
**Type of place of residence**							<0.001*
Rural	1242	41.21	430	14.27	1672	55.47	
Urban	1098	36.43	244	8.10	1342	44.53	
Total	2340	77.64	674	22.36	3014	100	
**Highest educational level**							<0.001*
Higher	118	3.92	20	0.66	138	4.58	
No education	199	6.60	39	1.29	238	7.90	
Primary	474	15.73	201	6.67	675	22.40	
Secondary	1549	51.39	414	13.74	1963	65.13	
Total	118	3.92	20	0.66	138	4.58	
**Source of drinking water**							<0.001*
Borehole/tank	247	8.20	93	3.09	340	11.28	
Bottled water	4	0.13	0	0.00	4	0.13	
Didn't specify	4	0.13	2	0.07	6	0.20	
Other	120	3.98	36	1.19	156	5.18	
Rainwater	2	0.07	0	0.00	2	0.07	
Spring/well/river/lake/stream	282	9.36	124	4.11	406	13.47	
Tap/piped	1681	55.77	419	13.90	2100	69.67	
Total	2340	77.64	674	22.36	3014	100	
**Type of toilet facility**							<0.001*
Bucket toilet	30	1.00	2	0.07	32	1.06	
Bush	1212	40.21	430	14.27	1642	54.48	
Composting toilet	4	0.13	3	0.10	7	0.23	
Didn't specify	2	0.07	0	0.00	2	0.07	
Flushing/hanging toilet	782	25.95	149	4.94	931	30.89	
Other	75	2.49	20	0.66	95	3.15	
Pit latrine	235	7.80	70	2.32	305	10.12	
Total	2340	77.64	674	22.36	3014	100	
**Household has: electricity**							<0.001*
No	1313	43.56	448	14.86	1761	58.43	
Yes	1027	34.07	226	7.50	1253	41.57	
Total	2340	77.64	674	22.36	3014	100	
**Household has: refrigerator**							<0.001*
No	1452	48.18	486	16.12	1938	64.30	
Yes	888	29.46	188	6.24	1076	35.70	
Total	2340	77.64	674	22.36	3014	100	
							<0.001*
**Religion**							
ELCIN	929	30.82	231	7.66	1160	38.49	
No religion	55	1.82	17	0.56	72	2.39	
Other	183	6.07	45	1.49	228	7.56	
Protestant Anglican	572	18.98	152	5.04	724	24.02	
Roman Catholic	487	16.16	167	5.54	654	21.70	
Seventh Day Adventist	114	3.78	62	2.06	176	5.84	
Total	2340	77.64	674	22.36	3014	100	
**Sharing toilet facilities with other households**							<0.001*
Don't know	1214	40.28	430	14.27	1644	54.55	
No	837	27.77	171	5.67	1008	33.44	
Yes	289	9.59	73	2.42	362	12.01	
Total	2340	77.64	674	22.36	3014	100	
**Wealth index**							<0.001*
Middle	521	17.29	153	5.08	674	22.36	
Poorer	505	16.76	166	5.51	671	22.26	
Poorest	457	15.16	193	6.40	650	21.57	
Richer	515	17.09	109	3.62	624	20.70	
Richest	342	11.35	53	1.76	395	13.11	
Total	2340	77.64	674	22.36	3014	100	
**Type of mosquito bed net(s) slept under last night**							0.001*
No net	2150	71.33	592	19.64	2742	90.98	
Richest	342	11.35	53	1.76	395	13.11	
Total	2340	77.64	674	22.36	3014	100	
**Type of mosquito bed net(s) slept under last night**							0.001*
No net	2150	71.33	592	19.64	2742	90.98	
Only treated nets	129	4.28	64	2.12	193	6.40	
Only untreated nets	61	2.02	18	0.60	79	2.62	
Total	2340	77.64	674	22.36	3014	100	
**Main language spoken in home**							<0.001*
Afrikaans	199	6.60	32	1.06	231	7.66	
Damara/Nama	381	12.64	83	2.75	464	15.39	
English	34	1.13	3	0.10	37	1.23	
Herero	277	9.19	59	1.96	336	11.15	
Lozi	140	4.64	91	3.02	231	7.66	
Other	58	1.92	36	1.19	94	3.12	
Oshiwambo	1002	33.24	247	8.20	1249	41.44	
Rukwangali	205	6.80	111	3.68	316	10.48	
San	44	1.46	12	0.40	56	1.86	
Total	2340	77.64	674	22.36	3014	100	
**Total children ever born**							0.029*
Up to 2	1285	42.63	401	13.30	1686	55.94	
3 or 4	680	22.56	161	5.34	841	27.90	
5 and above	375	12.44	112	3.72	487	16.16	
Total	2340	77.64	674	22.36	3014	100	
**Mother's age at first birth (in years)**							0.043*
<18	565	18.75	191	6.34	756	25.08	
18-26	1624	53.88	450	14.93	2074	68.81	
>26	151	5.01	33	1.09	184	6.10	
Total	2340	77.64	674	22.36	3014	100	
**Health insurance cover**							0.063**
No	2070	68.68	614	20.37	2684	89.05	
Yes	270	8.96	60	1.99	330	10.95	
Total	2340	77.64	674	22.36	3014	100	
**Current age of child (in months)**							0.004*
0-12	1556	51.63	489	16.22	2045	67.85	
13-24	784	26.01	185	6.14	969	32.15	
Total	2340	77.64	674	22.36	3014	100	
**Child's residency**							<0.001*
Lives elsewhere	364	12.08	33	1.09	397	13.17	
Lives with mother	1976	65.56	641	21.27	2617	86.83	
Total	2340	77.64	674	22.36	3014	100	
							<0.001*
**Drugs taken for intestinal parasites in last 6 months**							
No	1488	49.37	358	11.88	1846	61.25	
Yes	852	28.27	316	10.48	1168	38.75	
Total	2340	77.64	674	22.36	3014	100	

* Significant at a 5% level of significance

** Significant at a 10% level of significance

With respect to the maternal characteristics, out of the 674 children who had diarrhoea morbidity, majority were birthed by mothers who were aged 20-34 years old, with secondary education attainment and from the Kavango and Caprivi regions whose main language spoken in the house was Oshiwambo. Likewise, majority were living in poorer to poorest wealth indexed households in the rural areas, with no electricity and refrigerator, and whose source of drinking water was from a tap/piped source and using the bush field as their toilet facility, as shown in Table 2. In addition, majority were from households practicing the Evangelical Lutheran Church in Namibia (ELCIN) religion, sleeping with no mosquito bed nets, and not sure if their toilet facilities were being shared with other households. Furthermore, out of the 674 children who had diarrhoea morbidity, majority were less than 12 months old, had not taken drugs for intestinal parasites in the last 6 months, living with their mothers, and were birthed by mothers who had given birth to a maximum of two children with their first birth occurring when they were 18-26 years old with no health insurance cover, as shown in Table 2.

### Association examinations

From Tables 1 to 2, by means of all the p-values significant at a 5% level of significance with the exception of the breastfeeding status (p-value=0.066) and health insurance cover (p-value=0.063) that were significant at a 10% level of significance, the breastfeeding and maternal related characteristics can be concluded to have a significant association with child diarrhoea morbidity. All these characteristics with significant associations were included in the fitted multivariable log-binomial model and the subsequent results shown in Tables 3 to 4.

**Table 3:** Output from the fitted log-binomial model of breastfeeding and child diarrhoea morbidity

	Relative Risk	P-value	95% Confidence Interval
**Breastfeeding status**			
Currently breastfeeding	0.880	0.060**	0.770-1.005
Not currently breastfeeding (Ref)			

** Significant at a 10% level of significance

(Ref) = reference category

**Table 4:** Output from the fitted log-binomial model of maternal characteristics and child diarrhoea morbidity

	Relative Risk	P-value	95% Confidence Interval
**Mother's age (in years)**			
<20	1.516	0.239	0.758 - 3.029
20-34	1.169	0.650	0.597 - 2.288
35-44	1.080	0.827	0.543 - 2.145
45-54 (Ref)			
**Region**			
Caprivi	1.852	<0.001*	1.402 - 2.446
Erongo	0.561	0.007*	0.367 - 0.857
Hardap	0.447	<0.001*	0.277 - 0.720
//Karas	0.673	0.048*	0.455 - 0.996
Kavango	1.720	<0.001*	1.306 - 2.263
Khomas (Ref)			
Kunene	0.785	0.183	0.550 - 1.121
Ohangwena	0.957	0.787	0.695 - 1.317
Omaheke	0.869	0.441	0.607 - 1.243
Omusati	1.186	0.296	0.861 - 1.631
Oshana	0.692	0.093**	0.450 - 1.063
Oshikoto	0.861	0.412	0.601 - 1.232
Otjozondjupa	0.836	0.312	0.592 - 1.183
**Type of place of residence**			
Rural	1.414	<0.001*	1.230 - 1.627
Urban (Ref)			
**Highest educational level**			
Higher (Ref)			
No education	1.131	0.628	0.688 - 1.858
Primary	2.055	0.001*	1.348 - 3.132
Secondary	1.455	0.076**	0.962 - 2.202
**Source of drinking water**			
Borehole/tank	0.821	0.735	0.261 - 2.578
Bottled water	4.61e-06	0.960	2.41e-214 - 8.83e+202
Didn't specify (Ref)			
Other	0.692	0.537	0.215 - 2.225
Rainwater	4.61e-06	0.972	1.28e-300 - 1.65e+289
Spring/well/river/lake/stream	0.916	0.881	0.293 - 2.868
Tap/piped	0.599	0.375	0.192 - 1.862
**Type of toilet facility**			
Bucket toilet	1.50e+04	0.963	3.30e-175 - 6.79e+182
Bush	6.27e+04	0.958	1.39e-174 - 2.84e+183
Composting toilet	1.03e+05	0.956	2.26e-174 - 4.65e+183
Didn't specify (Ref)			
Flushing/hanging toilet	3.83e+04	0.960	8.47e-175 - 1.74e+183
Other	5.04e+04	0.959	1.11e-174 - 2.28e+183
Pit latrine	5.50e+04	0.959	1.21e-174 - 2.49e+183
**Household has: electricity**			
No	1.410	<0.001*	1.223 - 1.627
Yes (Ref)			
**Household has: refrigerator**			
No	1.435	<0.001*	1.234 - 1.669
Yes (Ref)			
**Religion**			
ELCIN	0.843	0.439	0.548 - 1.298
No religion (Ref)			
Other	0.836	0.474	0.512 - 1.366
Protestant Anglican	0.889	0.600	0.573 - 1.379
Roman Catholic	1.081	0.725	0.700 - 1.672
Seventh Day Adventist	1.492	0.089**	0.941 - 2.366
**Sharing toilet facilities with other households**			
Don't know (Ref)			
No	0.649	<0.001*	0.553 - 0.760
Yes	0.771	0.021*	0.618 - 0.961
**Wealth index**			
Middle	1.692	<0.001*	1.270 - 2.253
Poorer	1.844	<0.001*	1.389 - 2.447
Poorest	2.213	<0.001*	1.677 - 2.919
Richer	1.302	0.088**	0.961 - 1.763
Richest (Ref)			
**Type of mosquito bed net(s) slept under last night**			
Only treated nets	1.536	<0.001*	1.242 - 1.900
Only untreated nets	1.055	0.798	0.699 - 1.594
No net (Ref)			
**Main language spoken in home**			
Afrikaans	0.362	<0.001*	0.240 - 0.546
Damara/Nama	0.467	<0.001*	0.338 - 0.645
English	0.212	0.006*	0.069 - 0.645
Herero	0.458	<0.001*	0.324 - 0.648
Other (Ref)			
Rukwangali	0.917	0.569	0.681 - 1.235
Lozi	1.029	0.855	0.760 - 1.392
Oshiwambo	0.516	<0.001*	0.390 - 0.683
San	0.560	0.043*	0.319 - 0.983
**Total children ever born**			
Up to 2	1.034	0.720	0.861 - 1.243
3 or 4	0.832	0.093**	0.672 - 1.031
5 and above (Ref)			
**Mother's age at first birth (in years)**			
<18	1.164	0.043*	1.005 - 1.349
18-26 (Ref)			
>26	0.827	0.243	0.600 - 1.138
**Health insurance cover**			
No (Ref)			
Yes	0.795	0.060**	0.626 - 1.010
**Current age of child (in months)**			
0-12	1.252	0.003*	1.077 - 1.457
13-24 (Ref)			
**Child's residency**			
Lives elsewhere (Ref)			
Lives with mother	2.947	<0.001*	2.111 - 4.113
**Drugs taken for intestinal parasites in last 6 months**			
No (Ref)			
Yes	1.395	<0.001*	1.222 - 1.592

* Significant at a 5% level of significance

** Significant at a 10% level of significance

(Ref) = reference category

### Effect examinations

#### Breastfeeding related characteristics

From Table 3, with a significant p-value at a 10% level of significance, it can be concluded that the risk of diarrhoea morbidity for a child currently being breastfed (RR=0.880; p-value=0.060; 95% CI:0.770-1.005) was 0.88 times lower compared to the risk for a child who was not currently being breastfed.

#### Maternal related characteristics

From Table 4, with a significant p-value at a 5% -10% level of significance, it can be concluded that the risk of diarrhoea morbidity for a child whose mother was living in the Hardap (RR=0.447; p-value<0.001; 95% CI:0.277-0.720), Erongo (RR=0.561; p-value=0.007; 95% CI:0.367-0.857), //Karas (RR=0.673; p-value=0.048; 95% CI:0.455-0.996) and Oshana (RR=0.692; p-value=0.093; 95% CI:0.450-1.063) regions was between 0.45 and 0.69 times lower compared to the risk for a child whose mother was living in the Khomas region. However, the risk for a child whose mother was living in the Kavango (RR=1.720; p-value<0.001; 95% CI:1.306-2.263) and Caprivi (RR=1.852; p-value<0.001; 95% CI:1.402-2.446) regions was between 1.72 and 1.85 times higher. The risk for a child whose household was in the rural areas (RR=1.414; p-value<0.001; 95% CI:1.230-1.627) was 1.41 times higher compared to the risk for a child whose household was in the urban areas, while the risk for a child whose mother had secondary (RR=1.455; p-value=0.076; 95% CI:0.962-2.202) and primary (RR=2.055; p-value=0.001; 95% CI:1.348-3.132) education attainment was between 1.46 and 2.06 times higher compared to the risk for a child whose mother had higher education attainment.

Furthermore, the risk of diarrhoea morbidity for a child living in a household with no electricity (RR=1.410; p-value<0.001; 95% CI:1.223-1.627) and no refrigerator (RR=1.435; p-value<0.001; 95% CI:1.234-1.669) was between 1.41 and 1.44 times higher compared to the risk for a child living in a household that had both, while the risk for a child whose household was a Seventh Day Adventist devotee (RR=1.492; p-value=0.089; 95% CI:0.941-2.366) was 1.49 times higher compared to the risk for a child whose household was not religious, as shown in Table 4. With respect to the household's wealth index, the risk for a child who came from a richer (RR=1.302; p-value=0.088; 95% CL0.961-1.763), middle (RR=1.692; p-value<0.001; 95% CI:1.270-2.253), poorer (RR=1.844; p-value<0.001; 95% CI:1.389-2.447) and poorest (RR=2.213; p-value<0.001; 95% CI:1.677-2.919) wealth indexed household was between 1.30 and 2.21 times higher compared to the risk for a child who came from a richest wealth indexed household, while the risk for a child whose household did not share (RR=0.649; p-value<0.001; 95% CI: 0.553-0.760) and did share (RR=0.771; p-value=0.021; 95% CI:0.618-0.961) their toilet facilities with other households was between 0.65 and 0.77 times lower compared to the risk for a child whose household did not know if they shared their toilet facilities with other households. The risk for a child whose household slept in only treated mosquito bed nets the previous night (RR=1.536; p-value<0.001; 95% CI:1.242-1.900) was 1.54 times higher compared to the risk for a child whose household did not sleep in a mosquito bed net, while the risk for a child who lives with his/her mother (RR=2.947; p-value<0.001; 95% CI:2.111-4.113) was 2.95 times higher compared to the risk for a child who lives elsewhere.

Moreover, the risk of diarrhoea morbidity for a child whose mother's main language spoken in the house was English (RR=0.212; p-value=0.006; 95% CI:0.069-0.645), Afrikaans (RR=0.362; p-value<0.001; 95% CI:0.240-0.546), Herero (RR=0.458; p-value<0.001; 95% CI:0.324-0.648), Damara/Nama (RR=0.467; p-value<0.001; 95% CI:0.338-0.645), Oshiwambo (RR=0.516; p-value<0.001; 95% CI:0.390-0.683) and San (RR=0.560; p-value=0.043; 95% CI:0.319-0.983) was between 0.21 and 0.56 times lower compared to the risk for a child whose mother spoke other languages in the house, while the risk for a child who had taken intestinal parasites drugs in the last six months (RR=1.395; p-value<0.001; 95% CI:1.222-1.592) was 1.4 times higher compared to the risk for a child who had not, as shown in Table 4. The risk for a child whose mother gave birth to a total of three or four children (RR=0.832; p-value=0.093; 95% CI:0.672-1.031) was 0.83 times lower compared to the risk for a child whose mother gave birth to a total of more than four children, while the risk for a child whose mother had her first childbirth when she was less than 18 years old (RR=1.164; p-value=0.043; 95% CI:1.005-1.349) was 1.16 times higher compared to the risk for a child whose mother gave birth to her first childbirth at the age of 18-26 years old. However, the risk for a child who was aged 0-12 months (RR=1.252; p-value=0.003; 95% CI:1.077-1.457) was 1.25 times higher compared to the risk for a child who was aged 13-24 months, while the risk for a child whose mother had health insurance cover (RR=0.795; p-value=0.060, 95% CI:0.626-1.010) was 0.80 times lower compared to the risk for a child whose mother did not have.

## Discussion

In this study, the effect of breastfeeding and maternal characteristics on diarrhoea morbidity among children aged 0-2 years in Namibia was estimated using a multivariable log-binomial model. Out of the 674 children aged 0-2 years who had diarrhoea morbidity, approximately 355 were not currently breastfeeding. This high diarrhoea morbidity cases can be attributed to the inadequate water, sanitation and hygiene services at some of these households where the children were living and had their daily food prepared in sub-standard environment. This deduction is similar to the observations made in John Hopkins Medicine^2^, Centers for Disease Control and Prevention^4^, Getahun & Adane^7^ and UNICEF^8^, where it was highlighted that ‘child diarrhoea (morbidity) may be caused by many things including bacterial infection, viral infection, reaction to medicines, and even food intolerance, allergies and poisonings due to poor water, sanitation and hygiene in the affected households’.

Furthermore, diarrhoea morbidity was less likely to occur in children who were currently being breastfed, and whose mothers were living in the Hardap, Erongo, //Karas and Oshana regions in households whose main languages spoken in the house were English, Afrikaans, Herero, Damara/Nama, Oshiwambo and San. Likewise, the morbidity was less likely to occur in children whose mothers had health insurance cover and gave birth to a total of three or four children. However, the morbidity was more likely to occur in children aged 0-12 months who had taken intestinal parasites drugs in the last 6 months and lived with their motherin rural area households within the Kavango and Caprivi regions with no electricity and refrigerator, and whose households were followers of the Seventh Day Adventist religion. Likewise, the morbidity was more likely to occur in children whose mothers had their first childbirth when they were less than 18 years old with primary and secondary education attainment, and from poorest to middle wealth indexed households. These findings are not surprising. Firstly, children who were still breastfeeding were primarily being fed breast-milk which contains antibodies that helps protect against many common childhood illnesses while providing all the energy and nutrients that the children need, especially, during the first months of their lives^14^. However, the current health statuses and underlining illnesses of the breastfeeding mothers can contaminate the quality of the breastmilk during feeding, especially mothers living with infectious illnesses such as the human immunodeficiency virus, hepatitis B virus, hepatitis C virus, cytomegalovirus, West Nile virus and human T-cell lymphotropic virus.

Secondly, the high risk of child diarrhoea morbidity in the Kavango (now called Kavango East and Kavango West) and Caprivi (now called Zambezi) regions can be linked to the high spread of informal settlements inhabitants in these (still-developing) regions, with majority of the inhabitants migrating from other regions in search of work and a chance of having a decent and better standard of living, similar to the findings in Bauleth^9^, where it was concluded that ‘residents from informal settlements and those living in houses with walls made up of corrugate irons were significantly affected by diarrhoea, with such living conditions reported to create a high risk for water-borne and gastrointestinal diseases including diarrhoeal diseases’. Likewise, the high risk of diarrhoea morbidity in children aged 0-12 months, who lived with their mothers in rural area households and gave birth to their first child when they were below 18 years old can be attributed to negligence on the part of the mothers of the children due to their current employment status, working hours of their occupation, studies and financial means as well as the (poor) standards and environments of the mothers' households. Additionally, the high risk of diarrhoea morbidity in children living in rural area households with no electricity and refrigerator, and were followers of the Seventh Day Adventist religion is mostly as a result of these mothers believing in their traditional/religious beliefs/faiths to heal them more than modern day/scientific medicines whenever any of their household members are sick or alienated with diseases/illnesses. Also, electricity and refrigerators are luxuries that most households, if not all, in the rural areas cannot afford to own or buy due to their financial and employment statuses.

Moreover, the low risk of diarrhoea morbidity in children whose mothers were living in the Hardap, Erongo, //Karas and Oshana regions in households whose main languages spoken in the house were English, Afrikaans, Herero, Damara/Nama, Oshiwambo and San can be attributed to the fact that these regions are urbanized and well-developed with good accesses to health care and welfare facilities. This finding also concurs with Moreira de Sousa^13^, where it was concluded that urban areas were somewhat less likely to have a higher diarrhoea risk. Also, majority of the sponsored health information meant to raise awareness on healthy societal issues were most often written and translated in the English, Afrikaans, Herero, Damara/Nama, Oshiwambo and San languages that are already familiar to most communities in Namibia. Similarly, the low risk of diarrhoea morbidity in children whose mothers gave birth to a total of three or four children can be attributed to the fact that non-first-time mothers tend to have (more) life experiences in taking care of (under-five) children compared to first-time mothers.

## Conclusions

With the breastfeeding and maternal related characteristics such as the child's breastfeeding status, sharing toilet facilities with other households, total children ever born, health insurance cover and main language spoken in home havin less risks on child diarrhoea morbidity, while characteristics such as type of place of residence, highest educational level, electricity and refrigerator in the household, religion, wealth index, type of mosquito bed net(s) slept under last night, mother's age at first birth, current age of child, child's residency and drugs taken for intestinal parasites in last 6 months had more risks and region had mixed risks, it can be recommended that the Namibian government and policy makers continue to put more efforts in enforcing the ‘WHO and UNICEF recommendations of initiating exclusive breastfeeding for the first six months of the child's life and from the age of six months, introduce nutritionally-adequate and safe complementary (solid) foods together with continued breastfeeding up to two years of age or beyond’, while keeping the health statuses of the mothers in mind. This can be done through MOHSS increasing the free breastmilk given to new mothers from the usual three months of the child's birth to at least six months, and the breastmilk supplies can be sourced voluntarily from healthy women and mothers in the maternal age range in the country through the introduction of non-profit breastmilk-bank initiatives, similar to the national blood donation initiatives. This will help new mothers, especially those suffering from lactation failure and low milk production supply during breastfeeding as well as those with underlining illnesses that can contaminate the quality of the breastmilk during feeding.

In addition, since studies have shown that the possibility of reducing the risk of morbidity and mortality related to diarrhoeal infections in children requires well-informed parents, all relevant organizations and governmental ministries that deals with health services and children's well-being and protection should make use of mass media like radio and television to constantly spread consistent messages on breastfeeding and advocate for better implementation of sanitation and hygiene practices among mothers with children aged 0-2 years, especially in the Kavango (now called Kavango East and Kavango West) and Caprivi (now called Zambezi) regions to reduce the occurrence of diarrhoeal morbidity and diseases. This can be achieved through constantly engagements and collaborations with community-based organizations such as churches, traditional authorities, community leaders, local authorities and regional councils to assist with the translations of relevant information and communications in all the different languages spoken in the country, especially the indigenous ones spoken in regions such as Kavango (now called Kavango East and Kavango West) and Caprivi (now called Zambezi). Moreover, further studies on this topic is recommended using data from the next NDHS, pending available funding from USAID and other sponsors.

## Study limitations

This study was prone to recollection bias since majority of the questions in the 2013 NDHS questionnaire relied on memory recollection from the participating mothers which could have led to the possibilities of systematic under-reporting of some family sociodemographic characteristics and information. Although young children often have more than three loose/watery stool/bowel movements, even without diarrhoeal related morbidity, the stooling information collected from the mothers in the NDHS did not make this clear distinction, but instead classified the stooling information into diarrhoea without blood and diarrhoea with blood after asking the mother whether there was blood in the child's stools. Additionally, the NDHS information on the diarrhoeal history of the children were obtained based on the self-reporting responses from the participating mothers' recollection of the diarrhoeal history of their children within the last 2 weeks preceding the interview day(s) of the mothers. As such, children who had multiple diarrhoea morbidities prior to the last 2 weeks preceding the interview day(s) of their mothers would have been classified as having no diarrhoea morbidity in the NDHS. Furthermore, being a cross-sectional study design, it is possible that some participating mothers could have restarted breastfeeding of their children when they were sick of diarrhoea, after a long while of not breastfeeding.
